# Serum pepsinogens as a gastric cancer and gastritis biomarker in South and Southeast Asian populations

**DOI:** 10.1371/journal.pone.0230064

**Published:** 2020-04-09

**Authors:** Muhammad Miftahussurur, Langgeng Agung Waskito, Hafeza Aftab, Ratha-korn Vilaichone, Phawinee Subsomwong, Iswan Abbas Nusi, Ari Fahrial Syam, Thawee Ratanachu-ek, Dalla Doohan, Gontar Siregar, Yudith Annisa Ayu Rezkitha, Kartika Afrida Fauzia, Varocha Mahachai, Yoshio Yamaoka

**Affiliations:** 1 Division of Gastroenterohepatology, Department of Internal Medicine, Faculty of Medicine-Dr. Soetomo Teaching Hospital, Universitas Airlangga, Surabaya, Indonesia; 2 Institute of Tropical Disease, Universitas Airlangga, Surabaya, Indonesia; 3 Department of Environmental and Preventive Medicine, Oita University Faculty of Medicine, Yufu, Japan; 4 Department of Gastroenterology, Dhaka Medical College and Hospital, Dhaka, Bangladesh; 5 Gastroenterology Unit, Digestive Diseases Research Center (DRC), Thammasat University Hospital, Pathumthani, Thailand; 6 Department of Medicine, Chulabhorn International College of Medicine (CICM), Thammasat University, Pathumthani, Thailand; 7 Division of Gastroenterology, Department of Internal Medicine, Faculty of Medicine, University of Indonesia, Jakarta, Indonesia; 8 Department of Surgery, Rajavithi Hospital, Bangkok, Thailand; 9 Division of Gastroenterohepatology, Department of Internal Medicine, Faculty of Medicine, University of Sumatera Utara, Medan, Indonesia; 10 Faculty of Medicine, University of Muhammadiyah Surabaya, Surabaya, Indonesia; 11 GI and Liver Center, Bangkok Medical Center, Bangkok, Thailand; 12 Department of Medicine, Gastroenterology and Hepatology Section, Baylor College of Medicine, Houston, TX, United States of America; 13 Global Oita Medical Advanced Research Center for Health, Yufu, Japan; Sapporo Ika Daigaku, JAPAN

## Abstract

Serum pepsinogens have been widely acknowledged as gastric mucosal biomarkers; however, a multicountry report on the benefits of pepsinogens as biomarkers has not yet been published. We analyzed 1,206 sera and gastric mucosal samples collected from Bangladesh, Bhutan, Indonesia, Myanmar, Nepal and Thailand then assessed the association between gastric mucosal changes and *Helicobacter pylori* infection. The new cutoff values for serum pepsinogen values were evaluated using a receiver operating characteristic analysis. The participants with *H*. *pylori* infection had significantly lower pepsinogen I and higher pepsinogen II values, but a lower pepsinogen I/II ratio than participants without the infection (all *P* < .001). The pepsinogen I and pepsinogen I/II values were significantly higher and lower, respectively, in individuals with atrophic gastritis than in those without (both *P* < .001). Among uninfected individuals, only the pepsinogen I/II ratio was significantly lower in atrophic individuals. Pepsinogen I/II ratio also were significantly different between disease among *H*. *pylori*-positive and *H*. *pylori*-negative individuals, suggesting the pepsinogen I/II ratio is a robust biomarker for determining both chronic and atrophic gastritis. The cutoffs for detecting chronic and atrophic gastritis for the pepsinogen I/II ratio were 4.65 and 4.95, respectively. In conclusion, pepsinogen levels are useful biomarker for both chronic gastritis and atrophic gastritis, but they should be used with caution. Population-based validation is necessary to determine the best cutoff values. Among all pepsinogen values, the pepsinogen I/II ratio was the most reliable gastric mucosal-change biomarker.

## Introduction

After the discovery of *Helicobacter pylori*, the bacteria that induce progressive gastric mucosal inflammation, a clearer explanation of the pathogenesis of gastric cancer was established. Long-term *H*. *pylori* infection induces a multistep histological cascade, starting with chronic gastritis, and progressing to atrophic gastritis, then changing to intestinal metaplasia and ultimately leading to adenocarcinoma [1, 2]. Based on that pathogenesis cascade, the loss of gastric glandular cells in chronic atrophic gastritis is an important precursor lesion, which commonly leads to gastric adenocarcinoma. The gold standard to determine gastric atrophy is histological examination, given that it can diagnose the grade of gastric mucosal atrophy as well as the topographical distribution [3]. However, histological examinations have some limitations, including the need for endoscopy, the difficult process of obtaining the gastric specimens, and the need for an expert pathologist. Therefore, cheaper, more convenient, and simpler serum pepsinogens (PGs) are a useful alternative for atrophic gastritis and gastric cancer screening [4].

PGs originating from the gastric mucosa can be classified into two immunochemically distinct groups: pepsinogen I (PGI); and pepsinogen II (PGII), which are mostly secreted into the gastric lumen, with only 1% of total PGs circulating in the blood [5]. PGI is mainly secreted by mucosal cells in the fundus, whereas PGII is mainly secreted by chief cells, the proximal duodenal mucosa, and the pyloric glands [6]. In terms of screening for atrophic gastritis, an increasing PGII level and a decreasing PG I/II ratio have recently been associated with the grade of inflammation [7, 8]. These data suggest an opportunity for the application of PG levels when assessing patients with chronic gastritis [9]. It is important to note that certain variables, such as race, age, sex, and *H*. *pylori* infection, also influence PG levels [10–12]. Thus, discriminative baseline PG levels might vary by country of origin and should be recalculated, given that several factors, including geographic area, also contribute to these levels. In combination with *H*. *pylori* infection status as determined by IgG anti-*H*. *pylori* testing, ABC method is widely used to classify the high and low risk for developing gastric cancer based on the combination of pepsinogens values and the *H*. *pylori* status*H*. *pylori*-negative/PG-negative (group A); *H*. *pylori*-positive/PG-negative (group B); *H*. *pylori*-positive/PG-positive (group C); and *H*. *pylori*-negative/PG-positive (group D) [4]. The high-risk groups for developing gastric cancer are groups C and D; PG-positive group as the marker of the presence of atrophic gastritis [4]. Age-standardized incidence rates (ASR) of gastric cancer are 5.3/100,000 and 5.7/100,000 for South Asia and Southeast Asia, respectively (GLOBOCAN 2018 [available from: http://gco.iarc.fr/]). South Asia includes Bangladesh and Nepal, which are considered countries at low risk of gastric cancer, with ASRs of 5.2 and 6.3 per 100,000 population, respectively (GLOBOCAN 2018); as well as Bhutan, which is considered a high-risk country, with an ASR of 19.2/100,000 population. On the other hand, Southeast Asia includes Indonesia and Thailand, which are considered to be at low risk (ASR 1.2 and 3.6 per 100,000 population, respectively); as well as Myanmar, which is an intermediate-risk country, with an ASR of 12.8/100,000 population.

Serum pepsinogen level had been reported to have many benefits in several populations, including populations in Asia. Serum pepsinogen was useful for diagnostic tools of gastric cancer in India [13]. The similar benefit for gastric cancer screening was also reported in Bangladesh, but validation is required [14]. Additionally, the serum PGI/II ratio also had a significant association to the increasing risk of gastric cancer in Korean population [15]. In addition to the benefit for the gastric cancer screening tools, serum pepsinogen was also had benefit in the detecting *H*. *pylori* eradication status and gastric acidity. It is reported the PGI and PGI/II ratio was showing good accuracy for determining gastric acidity status ranging from 80–93% of analysed case. This benefit was improved after stratification of the *H*. *pylori* infection status [16]. The PG values, especially PGI/II ratio was increased in the case of succesful treatment of H. pylori eradication with an excellent diagnostic accuracy, suggesting the recovery of gastric mucosal condition after eradication therapy. These data showing the benefit of serum PG values in several population, however those are only showing in only one contry. There is no data in the multicountries approach. In this study, we analyzed the PG levels across participants from six countries in South and Southeast Asia. We also calculated the best cutoff and predictive values for discriminating chronic and atrophic gastritis based on PG levels.

## Materials and methods

### Study population

We conducted a cross-sectional study and enrolled patients to undergo an endoscopy examination on October to December 2014 in Surabaya, Indonesia; on February 2016 in Medan, Indonesia; on January 2016 in Chiang Kong and Chiang Saen, Thailand; on August 2015 in Ranong, Thailand; and on November 2014 in Dhaka, Bangladesh. Additionally, we used PG data from Nepal, Myanmar, and Bhutan from our previous studies [17–19]. Our exclusion criteria were individuals with a history of partial or total gastrectomy, nonfasted individuals, and those with a contraindication for upper endoscopy examination. Our ethical committee in each country only allowed us to take maximum two biopsies in cases of absence of gastric cancer. During the endoscopic examination procedure, we obtained two gastric biopsy specimens; one from the lesser curvature of the antrum, approximately 3 cm from the pyloric ring, and another from the greater curvature of the corpus. Those two specimens were used for the histological examination. The clinical outcome for peptic ulcer diseases (PUD) and gastric adenocarcinoma was determined by endoscopic examination and was confirmed by histological examination. Gastritis was determined based on the histological examination. We also collected fasting serum on the day of the endoscopy, and the samples were stored at −20°C. We obtained written informed consent from all participants prior to study participation. The study protocol was approved by the ethics committees of the Dr. Soetomo Teaching Hospital (Surabaya, Indonesia), the H. Adam Malik Hospital (Medan, Indonesia), the Thammasat University (Pathum Thani, Thailand), the Bangladesh Medical Research Council (Dhaka, Bangladesh), and the Oita University Faculty of Medicine (Yufu, Japan).

### Determination of *H*. *pylori* serology and PG levels

We separated the collected serum to measure *H*. *pylori* antibody titers and PG levels. We then measured the anti-*H*. *pylori* IgG levels with an anti-*H*. *pylori* enzyme-linked immunosorbent assay (ELISA) kit (Eiken, Co. Ltd., Tokyo, Japan), whereas the PGI and PGII levels were measured with a PG ELISA kit (Eiken, Co. Ltd.), following the manufacturer’s recommendation. We considered participants were infected with *H*. *pylori* when the serum *H*. *pylori* antibody titers were ≥10 U/mL, as recommended by the manufacturer. In addition, we classified the participants with a PGI/II ratio ≤3.0 and PGI <70 ng/mL as PG positive, according to the criteria of Miki [4]. According to those two values, we classified the participants into four groups: *H*. *pylori*-negative/PG-negative (group A); *H*. *pylori*-positive/PG-negative (group B); *H*. *pylori*-positive/PG-positive (group C); and *H*. *pylori*-negative/PG-positive (group D), as previously described and known as the ABC method [4].

### Histology and immunohistochemistry

All the biopsy specimens were fixed in 10% buffered formalin prior to the histology examination and then were embedded in paraffin for section cutting. The cut sections were stained with hematoxylin-eosin (HE) and May–Giemsa to determine the degree of gastritis and the *H*. *pylori* infection status. The determination of inflammation, atrophy, degree of intestinal metaplasia (IM) and bacterial load density were classified into four grades according to the updated Sydney system as 0, normal; 1, mild; 2, moderate; and 3, marked [20]. We considered samples as positive for *H*. *pylori* when bacterial loads were ≥grade 1.

To increase the accuracy of *H*. *pylori* detection, we also performed an immunohistochemistry (IHC) examination, as previously described [21]. Briefly, after antigen retrieval and inactivation of endogenous peroxidase activity, tissue sections were incubated with anti-α-*H*. *pylori* antibody (DAKO, Glostrup, Denmark) overnight at 4°C. After washing, the sections were incubated with biotinylated goat anti-rabbit IgG (Nichirei Co., Tokyo, Japan), followed by incubation with an avidin-conjugated horseradish peroxidase solution (Vectastain Elite ABC Kit; Vector Laboratories Inc., Burlingame, CA, USA). Peroxidase activity was detected using an H_2_O_2_/diaminobenzidine substrate solution. To minimize potential bias, the same experienced pathologist (TU) who also performed the experiments for Myanmar, Vietnam, Bhutan, Dominican Republic, and Indonesia [22–27] evaluated all the specimens in this study.

The topographically predominant gastritis was determined as follows: antral-predominant gastritis was considered when the atrophic score in the antrum was greater than those atrophic scores in the corpus; corpus-predominant gastritis was considered when the atrophic score in the antrum was lower than atrophic scores in the corpus; pan-gastritis was considered when the atrophic score was equal between the antrum and the corpus [28–30].

### Gastric Cancer Risk Index and virulence factors

The Gastric Cancer Risk Index (GCRI) was assessed based on the modification of the original Meining’s GCRI [31] in the study by Tanaka, et al. [32]. Briefly, there are five categories of scoring, including antral atrophy, corporal atrophy, antral intestinal metaplasia (IM), corporal IM, and distribution of chronic gastritis. Mild, moderate, and severe atrophy both in the antrum and in the corpus were scored as 1, 2, and 3, respectively. The presence of IM was scored as 1. As for the distribution of gastritis, antral predominant gastritis, pan-gastritis, and corpus predominant gastritis were scored as 1, 2, and 3, respectively. The determination of *cagA* and vacuolating cytotoxin A (*vacA*) status was performed by polymerase chain reaction and sequencing, as previously described [33].

### Data analyses

Discrete variables were tested using Pearson’s chi-squared test; the ordinal class variables were tested with the Mann–Whitney *U*-test for two groups comparison and Kruskall-Wallis test for more than 2 groups comparison. Correlations between PG levels and gastric mucosal inflammation, atrophy, and *H*. *pylori* infection status were evaluated by Spearman’s rank coefficients (*r*). The normality of the continuous variables was evaluated by the Shapiro–Wilk normality test. Receiver-operating characteristic (ROC) curves were constructed to calculate the best cutoff values, including the area under the curve (AUC), positive predictive value (PPV), negative predictive value (NPV), and accuracy for discriminating *H*. *pylori* positivity, chronic gastritis, and atrophic gastritis. A multivariate analysis was performed to determine the odds ratio (OR) for the highest gastric cancer index among the countries, considering confounding factors including age, sex, and *H*. *pylori* infection. The entire statistical analysis was performed using SPSS statistical software package version 23.0 (SPSS Inc., Chicago, IL, USA).

## Results

### Pepsinogens and *H*. *pylori*

The newly enrolled participants were 540 individuals, consisting of 227 females and 313 males, with a median 44 years (range 13–92 years). The participants were from Surabaya (n = 70) and Medan (n = 40), Indonesia; Chiang Kong (n = 100), Chiang Saen (n = 46), and Ranong (n = 147), Thailand; and Dhaka (n = 130), Bangladesh. We also included previous PG data from Nepal (n = 146) [18], Myanmar (n = 252) [19], and Bhutan (n = 371) [22]. There were 91 participants with PUDs, and 12 participants had gastric cancer who were analyzed separately. All gastric cancer cases were advanced stage with adenocarcinoma. Finally, we analyzed 1,206 participants, comprising 548 females and 658 males, with a median age of 44 years (range 13–88 years). Among these 1,206 participants, we found 302 individuals with no neutrophils, monocyte infiltration, nor glandular atrophy on the antrum or corpus. Therefore, we defined them as normal individuals. We found that the age, PGI, PGII and PGI/II levels were not normally distributed.

**Table 1** shows the PG level based on sex and clinical outcome. We found that females had a higher median PGI level, PGII level, and lower PGI/II ratio, but not statistically significant. We found a significant positive correlation between age and PGI and PGII (r = 0.16 and r = 0.15, respectively, both *P* < .001). The normal individuals had significantly lower PGI levels than those with gastritis as well as individuals with PUD (53.1 ng/ml vs. 59.5 ng/ml and 66.6 ng/ml; *P* = .002 and *P* < .001, respectively). The PGII and PGI/II ratio of the normal individuals were significantly lower and higher, respectively, than were those of the individuals with other clinical diagnoses (all *P* < .001). Overall, PGI and PGII levels were significantly higher in individuals with *H*. *pylori* infection than in those without (61.8 ng/ml vs. 54.6 ng/ml and 17.9 ng/ml vs. 9.3 ng/ml, both *P* < .001, respectively), whereas PGI/II levels were significantly lower in individuals with *H*. *pylori* than they were in those without (3.5 vs. 5.7, *P* < .001). When we used PG level to determine *H*. *pylori* positivity, the cutoff value of PGII was 12.35 (sensitivity, specificity, PPV, NPV, and accuracy: 77.5%, 65.8%, 58.4%, 82.4%, and 70.2%, respectively), and the PGI/II ratio was 4.55 (sensitivity, specificity, PPV, NPV, and accuracy: 77.1%, 78.0%, 68.5%, 84.5%, and 77.6%, respectively) (**S1 Table**).

**Table 1:** The pepsinogen level between gender and *H*. *pylori* infection status.

Variables	N	PGI (ng/ml)	PGII (ng/ml)	PGI/II ratio
**Gender**				
Male	695	56.4	12.25	4.9
Female	614	59.8	12.85	4.7
**Clinical Outcome**				
Normal	302	53.1***	8.5**	6.0**
Gastritis	904	59.5	14.2	4.4
PUD	91	66.6	19.1	4.0
Gastric Cancer	12	69.3	16.0	4.4
***H*. *pylori* status**^‡^				
Positive	530	61.8*	17.9*	3.5*
Negative	779	54.6	9.3	5.7

Data are shown as median. PUD; Peptic Ulcer Disease

^‡^) Determined by histology and IHC

*) P < 0.001 between *H*. *pylori* negative and *H*. *pylori* positive

**) P < 0.01 between normal and other clinical diagnosis

***) P<0.001 between normal and gastritis as well as PUD

PG: Pepsinogen

**Fig 1:**
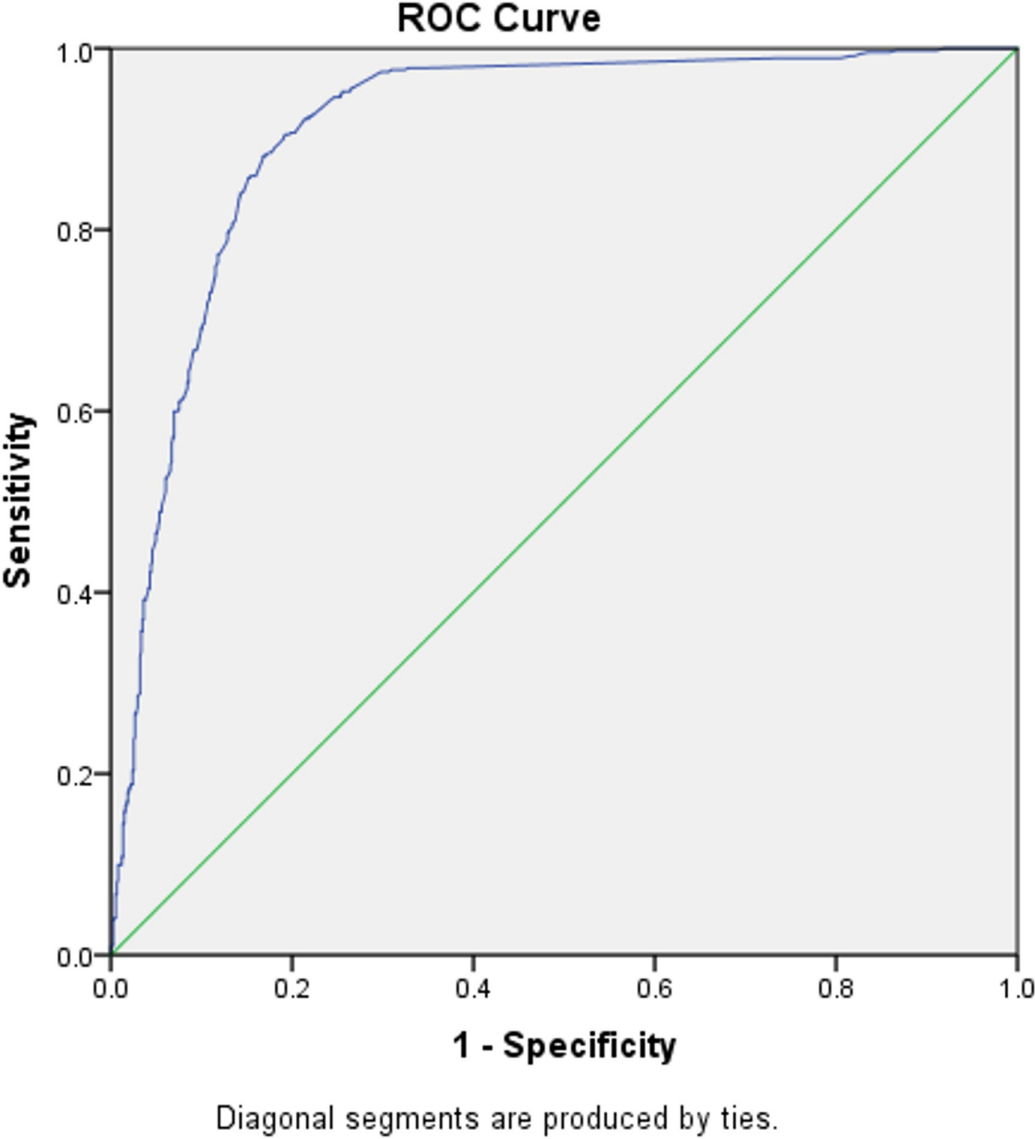
Receiver-operating characteristic (ROC) curve for determining the optimal cutoff of IgG for *H*. *pylori* infection compared to histology confirmed by IHC. Sensitivity, specificity, PPV, and NPV were 81.0%, 86.3%, 87.9%, and 78.6%, respectively.

By using the cutoff value according to the manufacturer’s recommendations (positive if ≥ 10 U/mL), we observed the sensitivity, specificity, PPV, and NPV of the ELISA kit IgG for *H*. *pylori* infection compared with the histology confirmed by IHC to determine positive and negative groups (81.0%, 86.3%, 87.9.0%, and 78.6%, respectively), with an AUC of 0.910 (95% confidence interval [CI] 0.893–0.927) (**Fig 1**). We observed that 105 (8.70%) patients had a past *H*. *pylori* infection, determined by the positive anti-*H*. *pylori* result, but a negative result of histology confirmed by IHC. We found no association between past infection status and PGI, PGII, and PGI/II ratio (*P* = .851, *P* = .672, *P* = .356, respectively).

### Pepsinogens and countries

**Fig 2:**
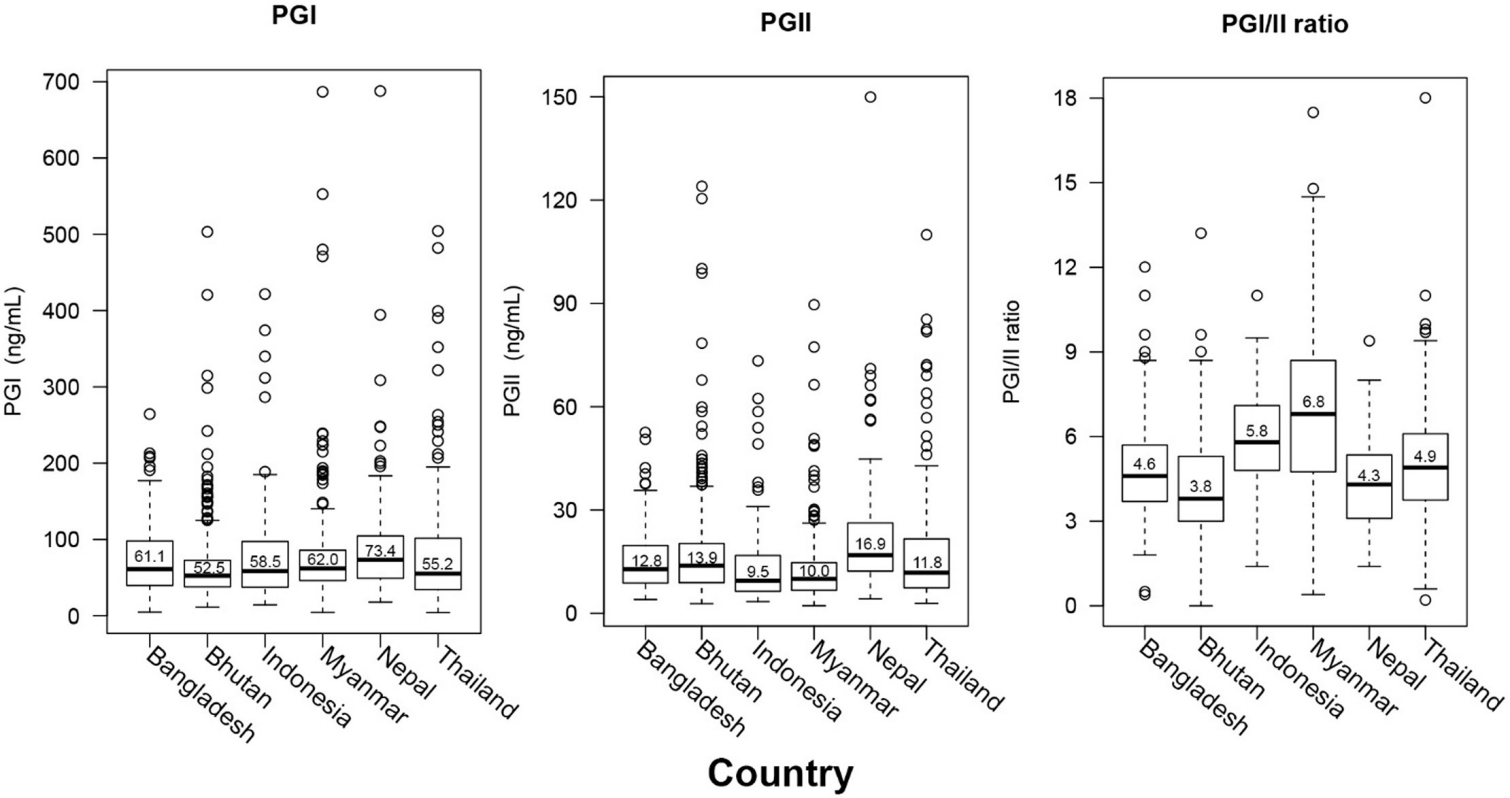
The pepsinogen level of *H*. *pylori* negative individuals by country. Bhutan was country with lowest PGI and PGI/II ratio values, Nepal was country with the highest PGII value.

Among the *H*. *pylori*-negative cases, we observed significant variations between country of origin and PGI, PGII, and PGI/II ratio (all *P* < .001, **Fig 2**). Individuals from Nepal had a higher median PGI value than did individuals from Bhutan and Thailand (73.4 ng/ml vs. 52.5 ng/ml and 55.2 ng/ml; *P* < .001 and *P* = .003, respectively). As for PGII values, individuals from Nepal had significantly higher values than those from Indonesia, Myanmar, Thailand, and Bangladesh (16.9 ng/ml vs. 9.5 ng/ml, 10.0 ng/ml, 11.8 ng/ml, and 12.8 ng/ml, respectively; all *P* < .001). Individuals from Myanmar had the highest median value of PGI/II, and it was significantly higher than that of individuals from other countries (6.8 vs. 3.8, 4.3, 4.6, 4.9, 5.8, and 6.8; all *P* < .001, **Fig 2**). The lowest median value of PGI/II was from Nepali participants, which was only significantly lower the median value of PGI/II of participants from Indonesia and Myanmar (both *P* < .001). Similar results were found among the individuals with *H*. *pylori* infection based on culture and histological examination. The lowest PGI level was among the participants from Bhutan, and individuals from Nepal had the highest PGII levels. PGI levels were significantly lower in individuals from Bhutan than they were in those from Myanmar and Thailand (56.9 vs. 69.2 and 79.2, *P* = .01 and *P* = .004, respectively; **S2 Table**).

### Pepsinogens and chronic gastritis

**Fig 3:**
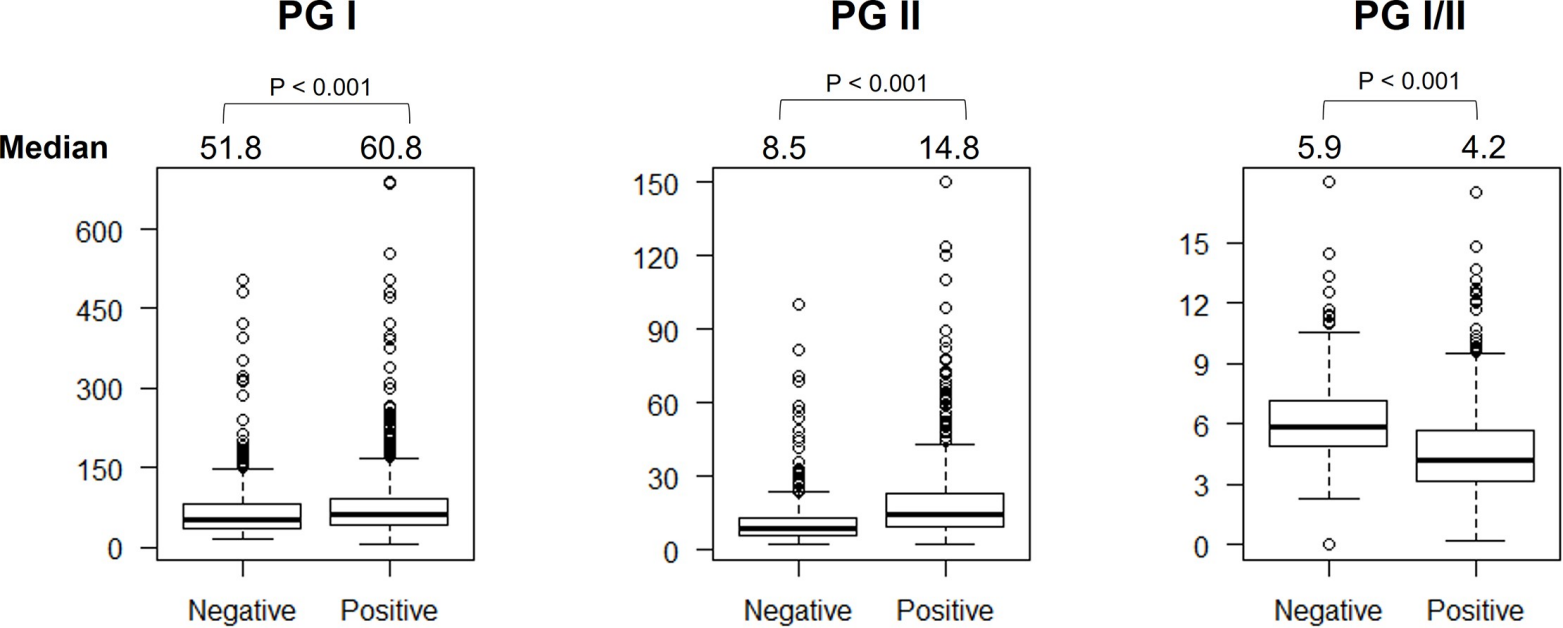
The pepsinogen level and chronic gastritis status. The PGI and PGII were significantly higher, whereas PGI/II ratio was significantly lower in the individuals with chronic gastritis than non-chronic gastritis one.

Chronic gastritis was defined as when monocyte infiltration was ≥1 regardless of the location. We found that PGI and PGII levels were significantly higher and PGI/II levels were significantly lower in patients with chronic gastritis than in those without it (60.8 vs. 51.8, 14.8 vs. 8.5, and 4.2 vs. 5.9, respectively; all *P* < .001, **Fig 3**). When we analyzed only the patients who were *H*. *pylori*-negative, we still observed the same trend in which PG I levels and the PG I/II ratio were significantly higher and lower in individuals with chronic gastritis than they were in those without (58.7 vs. 51.7 and 5.6 vs. 5.9, *P* = .04 and *P* = .01, respectively). There was no association between PG levels and predominant gastritis type.

When we considered a monocyte infiltration score ≥1 as positive for chronic gastritis, the best cutoff value of PG I/II for discriminating chronic gastritis was 4.85 (sensitivity, specificity, PPV, NPV, and accuracy: 63.4%; 75.6%; 83.9%; 50.8%; and 67.5%, respectively) with an AUC of 0.743 (95% CI 0.716–0.771) (**S1 Table**). When we used PG level as a test for moderate-severe gastritis, we classified those patients with an inflammation score >2 as the positive group and those without chronic gastritis as the negative group. The best cutoff value of PG I/II for identifying those with chronic gastritis was 4.65, with a sensitivity, specificity, PPV, and NPV of 76.9%, 84.1%, 81.2%, and 80.4%, respectively. The overall accuracy was 80.7%, with an AUC of 0.882 (95% CI 0.857–0.907) (**S1 Table**).

### Pepsinogens and atrophic gastritis

**Fig 4:**
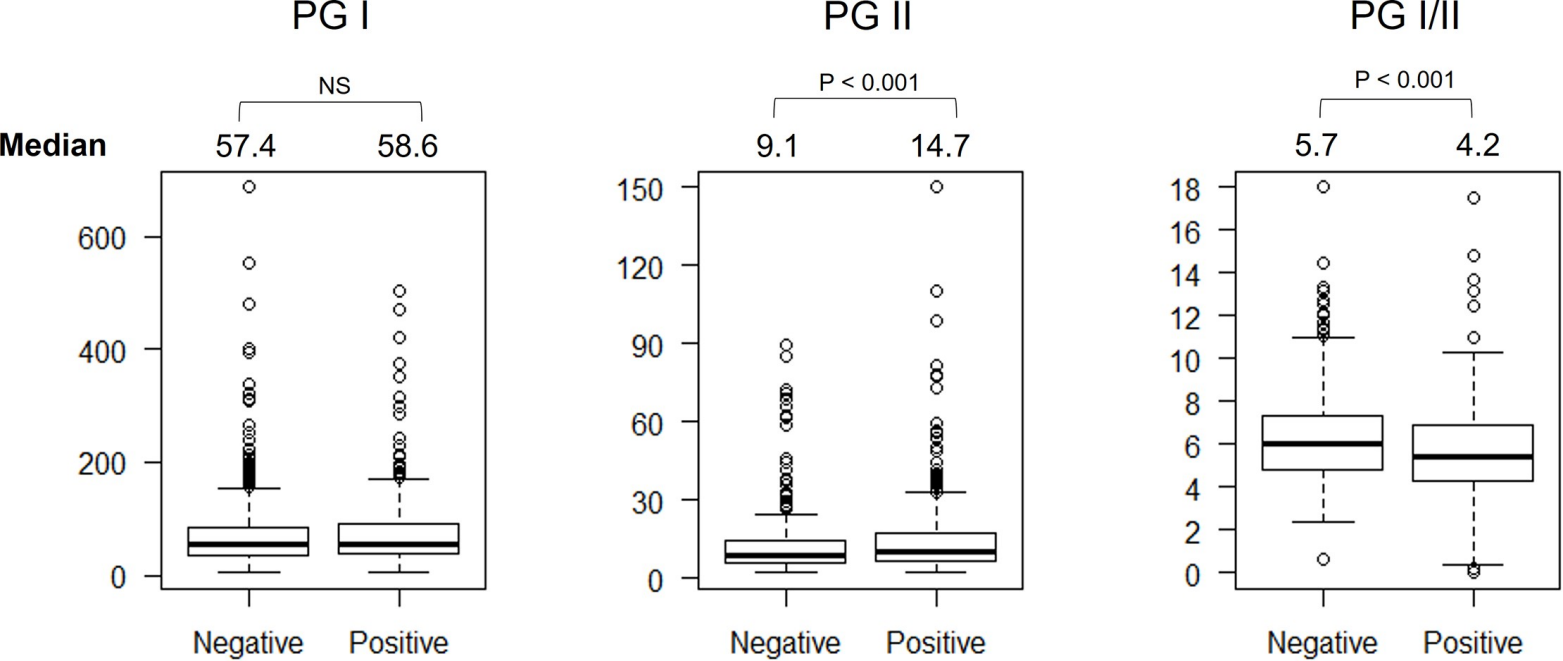
The pepsinogen level and gastritis atrophy status. The PGII was significantly higher and PGI/II ratio was significantly lower in the individuals with gastritis atrophy than non-gastritis atrophy one.

Compared to individuals with nonatrophic gastritis, those with atrophic gastritis had significantly higher PGII levels and lower PGI/II ratio (14.7 ng/ml vs. 9.1 ng/ml and 4.2 vs. 5.7; *P* < .001 and *P* = .001, respectively) (**Fig 4**). However, when we analyzed only *H*. *pylori-*negative samples, the PG I/II ratio was significantly lower in cases of atrophic than nonatrophic gastritis (6.0 vs. 5.4, *P* < .001). In addition, we observed a significant negative correlation between the PG I/II ratio and the degree of atrophy both in the antrum and corpus (*P* < .001, r = −0.13 and *P* < .01, r = −0.29, respectively). Finally, those patients with antral predominant atrophic gastritis had significantly lower PGII median values than those with panatrophic gastritis (*P* = .002, **Table 2**), whereas the PG I/II level of those with antral predominant atrophic gastritis had significantly higher median PGI/II ratio than those with corporal and panatrophic gastritis (both *P* < .001, **Table 2)**.

**Table 2:** The pepsinogen level by the predominant atrophic gastritis type.

Pepsinogen level (median)	Predominant Atrophic Gastritis		
	Antral	Corporal	Pangastritis
PGI (ng/ml)	57.4	52	60.7
PGII (ng/ml)	13.9*	16.2	18.0
PGI/II ratio	4.4**	3.2	3.4

*) Significantly lower pan-atrophic gastritis individuals

**) Significantly higher than corporal and pan-atrophic gastritis individuals

When we used the Miki criteria to define PG-positive status (PGI/II ratio ≤ 3.0 and PGI ≤ 70 ng/ml), we found that the overall prevalence of PG-positive individuals was very low (10.6%, 128 of 1206) compared with the histological observations (58.6%, 707 of 1,206). As expected, when we used both PG I/II ratios ≤3.0 and PGI ≤70 ng/ml for atrophic scores ≥1, the sensitivity, specificity, PPV, NPV, and accuracy were 15.9%, 96.9%, 89.3%, 44.9%, and 49.5%, respectively. Regarding the low sensitivity of the Miki criteria, we determined the best cutoff value of PGI, PGII, and the PG I/II ratio with a ROC curve analysis. The AUCs for the PGI, PGII, and PGI/II ratio discriminating an atrophy score ≥1 were 0.527 (95% CI 0.493–0.568), 0.664 (95% CI 0.633–0.696), and 0.718 (95% CI 0.689–0.747), respectively. The optimal cutoff values for the PGII and PGI/II ratio for discriminating an atrophy score ≥1 were 10.35 ng/mL for PGII (sensitivity 72.6% and specificity 56.9%, PPV 70.5%, NPV 59.4%, and accuracy 66.1%) and 4.95 for PGI/II (sensitivity 66.2%, specificity 67.5%, PPV 74.3%, NPV 58.5%, and accuracy 66.8%) (**S1 Table**).

**Fig 5:**
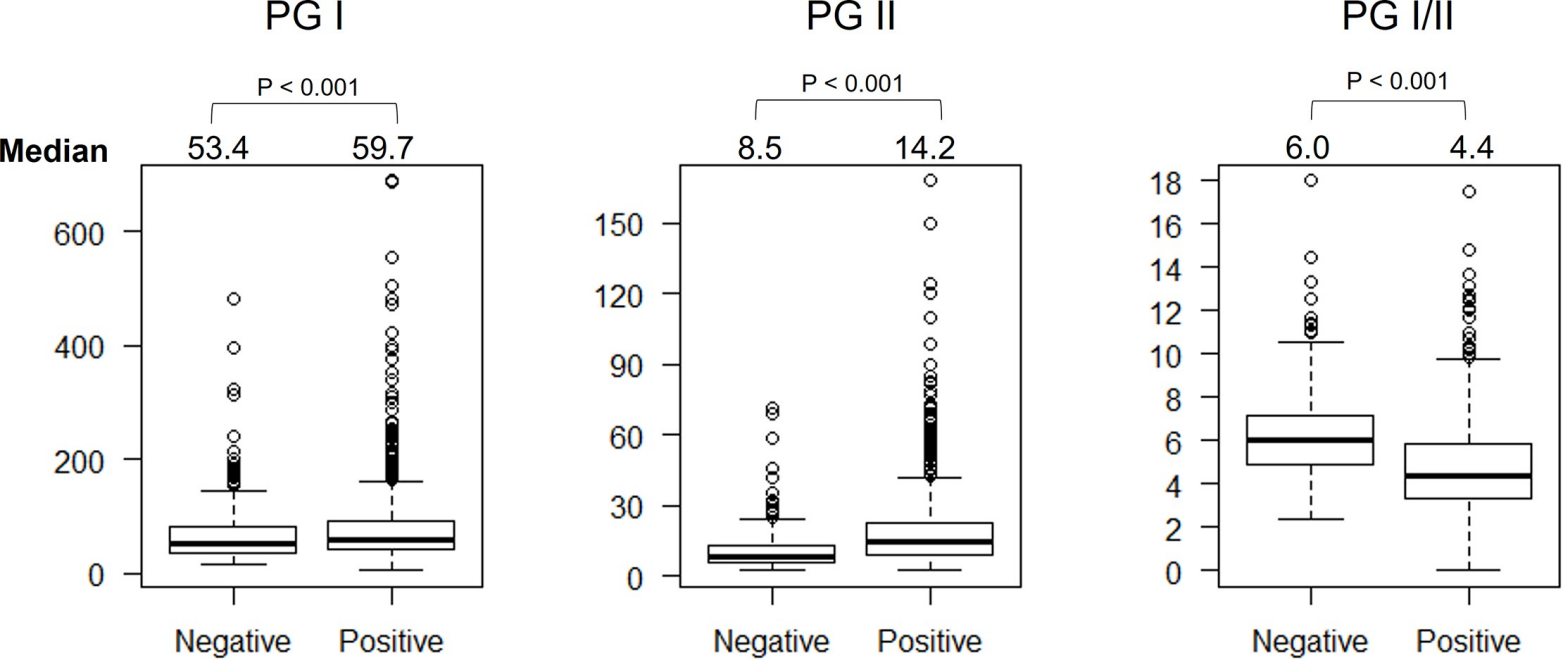
The pepsinogen level and the gastric abnormality. The gastric abnormality was assessed as any gastric mucosal changes either neutrophil and monocyte infiltration, gland atrophy or intestinal metaplasia. We observed the PGI and PGII was significantly higher and PGI/II ratio was significantly lower in individual with gastric abnormality than normal one.

Finally, we considered patients with any inflammation and atrophy as having an abnormal observation, and individuals without any abnormality were considered as normal individuals. We observed that PGI and PGII levels were higher and PG I/II ratios were lower among those patients observed with a gastric abnormality than they were among normal individuals (all *P* < .001) (**Fig 5**).

### Pepsinogens and the ABC method

A comparison of the ABC method between the countries is presented in **Table 3**. By using ≥10 U/mL for *H*. *pylori* infection status combined with PGI ≤70 ng/mL and a PGI/II ratio ≤3 for PG positivity, we determined the distribution of the four groups in each country according to the ABC method as proposed by Miki [4]. Overall, group A (683 of 1,206, 56.6%) was the most predominant, followed by group B (395 of 1,206, 32.7%), group C (94 of 1,206, 7.8%), and group D (34 of 1,206, 2.8%). There was a statistically significant difference in the distribution of the ABC classification between countries (*P* < .001). As the ABC group had an association to the incidence of AG, we calculate the OR of AG presence for each group. The odds of group B, C and D for AG were 6.67, 22.16 and 4.19 fold higher than group A, respectively (95%CI: 4.95–8.96; 9.55–51.38; 1.92–9.13, respectively).

**Table 3:** Distribution of ABC method by the countries.

Country	n	The ABC Classification (%)			
		Group A	Group B	Group C	Group D
Bangladesh	126	80 (63.5)	33 (26.2)	6 (4.8)	7 (5.6)
Bhutan	324	95 (29.3)	167 (51.6)	55 (16.9)	7 (2.2)
Indonesia	106	89 (83.9)	14 (13.2)	2 (1.9)	1 (0.9)
Myanmar	239	149 (62.3)	80 (33.5)	5 (2.1)	5 (2.1)
Nepal	128	71 (55.5)	42 (32.8)	11 (8.6)	4 (3.1)
Thailand	283	199 (70.3)	59 (20.9)	15 (5.3)	10 (3.5)

As expected, Bhutan had the highest proportion of group C individuals and the lowest proportion of group A individuals compared with those from other countries. In agreement with gastric cancer risk based on GLOBOCAN 2018 data, Indonesia (ASR 1.5/100,000) had the highest proportion of group A individuals and the lowest proportion of group C individuals (83.9% and 1.9%, respectively). Interestingly, Bangladesh had a considerably high percentage of group D individuals (5.6%), even though it was categorized as a low-risk gastric cancer country in GLOBOCAN 2018.

### The Pepsinogens, Gastric Cancer Risk Index, and virulence factor

There was no significant mean GCRI score difference between sexes (**Table 4**). We observed a significant positive and negative correlation between PGII level, PGI/II ratio, and GCRI index (r = 0.267 and r = −0.378, all *P* < .001, respectively). Bhutan had the highest GCRI index (mean [median] 2.4 [2]), and it was significantly higher than the rest of the countries (all *P* < .001). Bhutan, Nepal, and Bangladesh had higher odds of presenting a high GCRI index than did Indonesia (OR 8.55, OR 1.70, and OR 1.66, respectively). After adjusting for age, sex, and *H*. *pylori* infection status, Bhutan had a 6.9-fold higher odds of presenting a high GCRI index (95% CI 4.38–10.86, *P* < .001) than Indonesia.

**Table 4:** Gastric Cancer Risk Index among countries.

Characteristic	n	GCRI Score									Mean [median]
		1	2	3	4	5	6	7	8	9	
Total	1206	94	599	298	120	58	24	11	1	1	2.64 [2]
**Gender**											
Male	658	47	265	135	59	27	11	3	0	1	2.64 [2]
Female	548	47	334	163	61	31	13	8	1	0	2.65 [2]
**Country**											
Bangladesh	126	12	68	33	10	1	2	0	0	0	2.41 [2]
Bhutan	324	9	73	115	66	39	13	7	1	1	3.40 [3]
Indonesia	106	10	74	14	5	2	1	0	0	0	2.22 [2]
Myanmar	239	37	123	54	13	6	4	2	0	0	2.36 [2]
Nepal	128	10	72	33	9	2	1	1	0	0	2.43 [2]
Thailand	283	16	189	49	17	8	3	1	0	0	2.38 [2]

**Fig 6:**
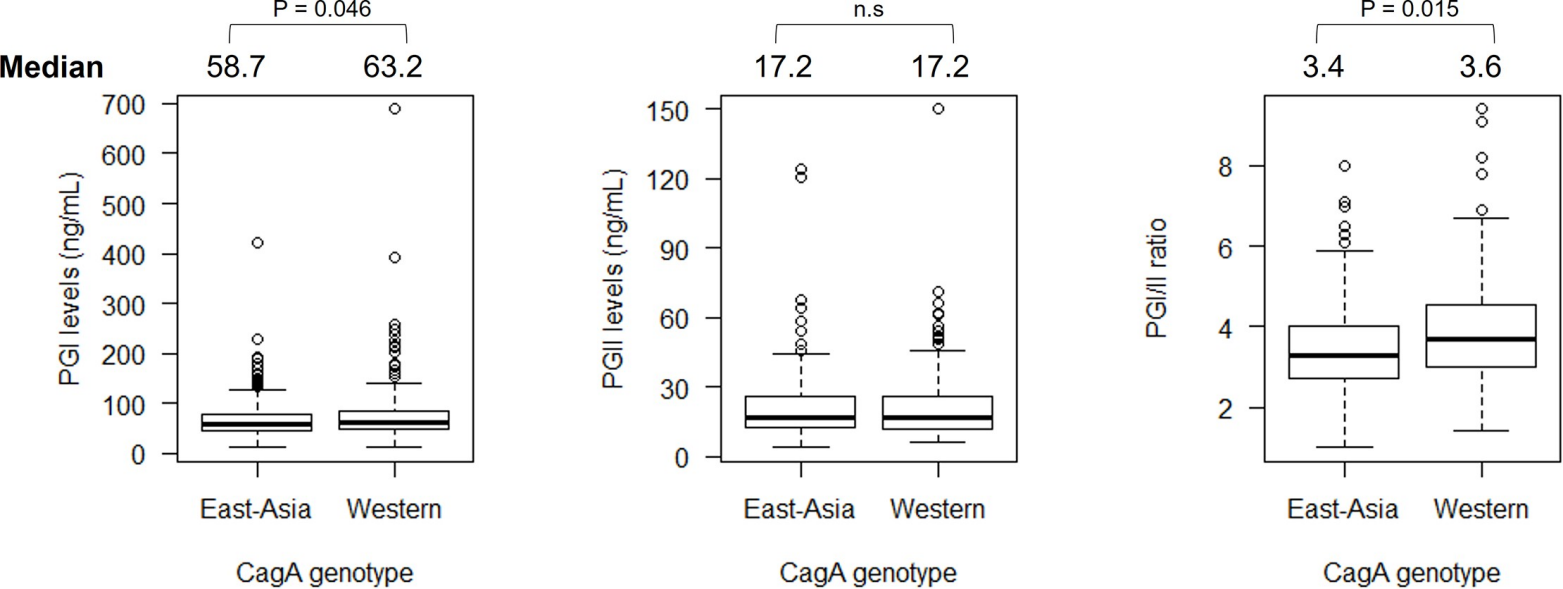
The association between pepsinogen levels between different CagA type. Individuals infected by western type CagA type showed significantly higher PGI than East-Asian one. Individuals infected by East-Asian CagA type were significantly lower PGI/II than western type one.

In addition, we analyzed the association between *H*. *pylori* virulence factors and PG levels. We observed that the distribution of the CagA type was different in each country. Bangladesh, Myanmar, Nepal, and Thailand were countries with predominantly Western-type CagA, whereas Bhutan and Indonesia were countries with predominantly East-Asian-type CagA (**Table 5**). We observed that PGI levels were higher in individuals with Western-type CagA than in those with the East-Asian-type CagA (63.2 vs. 58.7, *P* = .046), whereas the PGI/II ratio was significantly lower in those with East-Asian type CagA than in those with the Western-type CagA (3.4 vs. 3.6, *P* = .015) (**Fig 6**). As for the *vacA* genotype, we did not find any association between *vacA* genotype and PG levels (*P* > .05).

**Table 5:** Distribution of CagA type based on countries.

Country	n	CagA Type (%)	
		East-Asian type	Western type
Bangladesh	45	0 (0.0)	45 (100)
Bhutan	201	186 (92.5)	15 (7.5)
Indonesia	9	8 (88.8)	1 (11.1)
Myanmar	63	5 (7.9)	58 (92.0)
Nepal	41	3 (7.3)	38 (92.7)
Thailand	67	29 (43.2)	38 (56.7)

## Discussion

This study employed a multicountry approach to analyze the serum PG levels of individuals from six countries in the South Asia and Southeast Asia regions to assess correlations between PGs and gastroduodenal abnormalities. We also calculated the best cutoff values to predict the presence of chronic and atrophic gastritis based on PG levels. We observed that both PGI and PGII levels were significantly higher in the individuals infected with *H*. *pylori* than in those who were not. In addition, the PGI/II ratio of infected individuals was significantly lower than it was those patients who were not infected. The negative correlation of PGI/II with *H*. *pylori* positivity despite a positive correlation between both PGI and PGII with *H*. *pylori* infection was attributed to increasing PGII levels instead of decreasing PGI levels. This phenomenon was also supported by the higher correlation coefficient of PGII compared with that of PGI. Our finding is in concordance with that of previous studies which mentioned increasing PGII levels along with increasing severity of gastric mucosa inflammation [7, 34]. Additionally, increasing serum PGII was reported as a common observation with active *H*. *pylori* infection, which might be a result of hyperacidic conditions in the stomach [35]. In concordance with previous studies, our study also showed that increasing age was significantly associated with increased PGI and PGII levels [36, 37]. However, the increase in PG might not be affected by age alone, but might also be influenced by factors such as gastric inflammation and atrophic gastritis [37]. These findings showed the PG levels were strongly influenced by many factors including increasing age, gastric condition and even the different gender, suggesting the interpretation PG level need to consider those factors and adjustment by age and sex is necessary.

Our study showed that all PG levels, including PGI, PGII, and the PGI/II ratio were significantly different between individuals with and without chronic gastritis. However, when we analyzed only *H*. *pylori*-negative individuals, only PGI and PGI/II were significantly higher and lower in those patients with chronic gastritis than in those without it, respectively. These findings suggest that the PGI/II ratio might be a potential biomarker for determining chronic gastritis even in the absence of *H*. *pylori*, which is a main risk factor for chronic gastritis. This result is supported by that of a previous study that also reported that the PGI/II ratio was a useful biomarker for chronic gastritis [38]. Interestingly, the PGI/II ratio was more reliable in discriminating moderate-severe chronic gastritis than cases of mild gastritis, as shown by a better AUC value in moderate-severe chronic gastritis.

Our analysis of the association between PG values and atrophic gastritis showed that PGII and PGI/II were significantly affected by atrophic gastritis. However, when we analyzed only patients who were *H*. *pylori*-negative, only the PGI/II ratio showed a significantly lower result in patients with atrophic gastritis. This finding suggests that in determining atrophic gastritis cases, the PGI/II ratio might be a more reliable biomarker compared with PGI or PGII alone. Similar to this result, a previous study has also reported the reliability of the PGI/II ratio as an atrophic gastritis biomarker [39]. Spearman’s rank correlation test showed that there were significant negative correlations between the PGI/II ratio and the degree of atrophy; in other words, the higher the atrophic score, the lower the PGI/II ratio value. This result is in concordance with a previous study that reported that as the atrophic degree increases, the accompanying PGII increase will be more prominent while the PGI level will decrease, leading to a significant decrease in the PGI/II ratio [40, 41]. This finding suggested the most beneficial of PG in the detection of atrophic gastritis is the PGI/II ratio value.

We also analyzed gastric cancer risk by using the GCRI index. Our results showed that out of six countries, individuals from Bhutan and Indonesia had the highest and the lowest risk of developing gastric cancer, respectively. This finding was in concordance with the gastric cancer ASR reported in Bhutan of 24.2 in men and 13.5 in women per 100,000 population (GLOBOCAN 2018, http://gco.iarc.fr/). On the other hand, the ASR of gastric cancer in Indonesia was low (1.2/100,000 population), and was possibly the lowest worldwide (GLOBOCAN 2018, http://gco.iarc.fr/). Therefore, our study has shown that the GCRI index might be a valuable tool for assessing gastric cancer risk. However, a validation of the GCRI in various populations and geographical areas is still necessary.

*cagA* is widely known as one of the most important virulence factors for *H*. *pylori* and is strongly associated with the development of gastric mucosal inflammation [42, 43]. Our study revealed that patients infected with East Asian-type CagA had significantly lower PGI/II ratio compared with those infected by Western-type CagA. East Asian-type CagA has been reported to be more virulent than Western-type CagA due to greater binding to the Src homology 2 domain-containing protein tyrosine phosphatase 2 [44]; thus, it has ability to generate more severe gastric mucosal inflammation and is also associated with the development of atrophic gastritis and gastric cancer and it is described as lower PGI/II ratio value. Therefore, our result confirms the fact that the *cagA* genotype, especially the East-Asian type CagA, plays an important role for inducing more damage in the gastric epithelial cell of individuals infected with *H*. *pylori*.

Finally, we understand that PGI or PGII levels alone might not be sufficient to distinguish between gastric diseases, such as chronic gastritis and atrophic gastritis. The levels of PGI and PGII might be affected by many factors, such as race, age, sex, geographic area, and lifestyle [10, 12], resulting in inconsistent association with these diseases. However, our results have shown that the PGI/II ratio was significantly reduced in cases of gastric diseases such as chronic gastritis, atrophic gastritis, and *H*. *pylori* infection. Therefore, the PGI/II ratio might be a more reliable biomarker to help clinicians distinguish between various gastric diseases. In addition, even the PG levels had good advantage in the gastric cancer screening, our study population mostly dominated by gastritis case and the sample size is considerably low for the multi-country approach study. Therefore, further study with an increasing sample size, especially for gastric cancer case is necessary.

## Conclusions

PG showed benefits as a gastric mucosal change biomarker for gastric diseases, including chronic gastritis and atrophic gastritis, but it needs to be applied with caution. Population-based validation is necessary to determine the best cutoff points. Among all PG values, the PGI/II ratio appears to be the most reliable value as a gastric mucosal change biomarker.

## Supporting information

S1 Table Performance parameters of serum pepsinogen values to distinguish different disease groups. (DOCX)

S2 Table The characteristic of PG levels between countries on the *H*. *pylori* infected individuals. (DOCX)

## Abbreviations

ASR: Age-Standarization Rate
AUC: Area Under Curve
CagA: Cytotoxic antigen gene A
ELISA: Enzyme-linked Immunoabsorbent Assay
GCRI: Gastric Cancer Risk Index
GLOBOCAN: Global Cancer Incidence, Mortality and Prevalence
HE: Haematoxylin-Eosin
IHC: Immunohistochemistry
NPV: Negative Predictive Value
OR: Odds Ratio
PPV: Positive Predictive Value
PUD: Peptic Ulcer Disease
ROC: Receiver Operating Characteristic
VacA: Vacuolating Cytotoxic gene A
