# Supplementary material for: Serum pepsinogens as a gastric cancer and gastritis biomarker in South and Southeast Asian populations
Source: PLoS One. 2020 Apr 9;15(4):e0230064. doi: 10.1371/journal.pone.0230064 (PMC7145115; doi:10.1371/journal.pone.0230064)
Supplement: S1 Table — (DOCX) [file pone.0230064.s001.docx]

| **Disease group** | **Parameters** | **Serum Pepsinogens** | | |
| --- | --- | --- | --- | --- |
|  |  | **PGI** | **PGII** | **PGI/II** |
| *H. pylori* positive | Cutoff value | - | ≥ 12.35 ng/mL | ≤ 4.55 |
|  | Sensitivity | - | 77.5% | 77.1% |
|  | Specificity | - | 65.8% | 78.0% |
|  | PPV | - | 58.4% | 68.8% |
|  | NPV | - | 82.4% | 84.5% |
|  | Overall Accuracy | - | 70.2% | 77.6% |
| *Mild chronic gastritis* | Cutoff value | - | - | ≤ 4.85 |
|  | Sensitivity | - | - | 63.4% |
|  | Specificity | - | - | 75.6% |
|  | PPV | - | - | 83.9% |
|  | NPV | - | - | 50.8% |
|  | Overall Accuracy | - | - | 67.5% |
| *Moderate-Severe chronic gastritis* | Cutoff value | - | - | ≤ 4.65 |
|  | Sensitivity | - | - | 76.9% |
|  | Specificity | - | - | 84.1% |
|  | PPV | - | - | 81.2% |
|  | NPV | - | - | 80.4% |
|  | Overall Accuracy | - | - | 80.7% |
| *Atrophic gastritis* | Cutoff value | - | ≥ 10.35 ng/mL | ≤ 4.95 |
|  | Sensitivity | - | 72.6% | 66.2% |
|  | Specificity | - | 56.9% | 67.5% |
|  | PPV | - | 70.5% | 74.3% |
|  | NPV | - | 59.4% | 58.5% |
|  | Overall Accuracy | - | 66.1% | 66.8% |

**S1 Table. Performance parameters of Serum Pepsinogen Values to distinguish different disease groups.**
