# Supplementary material for: Serum pepsinogens as a gastric cancer and gastritis biomarker in South and Southeast Asian populations
Source: PLoS One. 2020 Apr 9;15(4):e0230064. doi: 10.1371/journal.pone.0230064 (PMC7145115; doi:10.1371/journal.pone.0230064)
Supplement: S2 Table — (DOCX) [file pone.0230064.s002.docx]

**S2 Table. The Characteristic of PG levels between countries on the *H. pylori* infected individuals.**

| **Characteristic** | **Countries** | | | | | |
| --- | --- | --- | --- | --- | --- | --- |
|  | **Bangladesh** | **Bhutan** | **Indonesia** | **Myanmar** | **Nepal** | **Thailand** |
| Total | 51 | 189 | 14 | 83 | 45 | 80 |
| Gender |  |  |  |  |  |  |
| Male (%) | 24 (47.0) | 112 (59.2) | 4 (28.5) | 34 (40.9) | 15 (33.3) | 48 (60) |
| Female (%) | 27 (53.0) | 77 (40.8) | 10 (72.5) | 49 (59.1) | 30 (66.7) | 32 (40) |
| Mean Age (±SD) | 36.3 ± 11.7 | 37.6 ± 13.6 | 56.2 ± 14.6 | 42.0 ± 12.4 | 41.4 ± 14.2 | 53.3 ± 12.8 |
| PG I (ng/mL) | 57.7 | 56.9 | 74.1 | 69.2 | 73.4 | 79.2 |
| PG II (ng/mL) | 16.1 | 17.1 | 21.3 | 14.1 | 25.9 | 24.3 |
| PG I/II | 3.9 | 3.3 | 3.9 | 4.7 | 3.1 | 3.4 |
